# New methods are revolutionizing biology: an interview with Martin Steinegger

**DOI:** 10.1093/nsr/nwaf142

**Published:** 2025-04-11

**Authors:** Weijie Zhao

## Abstract

In the 2021 *Nature* paper [1] that introduced the groundbreaking protein structure prediction artificial intelligent (AI) tool AlphaFold2 to the world, Martin Steinegger was the only author who was not affiliated with DeepMind. At the forefront of the ongoing revolution in biological research methodology, Steinegger has pioneered the development of a series of powerful bioinformatics tools, including MMseqs2 [2] for protein and nucleotide sequence searching and clustering, Foldseek [3] for protein structure searches at the scale of the AlphaFold database and ColabFold [4] for rapid and accessible protein structure prediction.

After earning his PhD in Computer Science with summa cum laude honors from the Technical University of Munich in 2018, based on his doctoral research that was conducted at the Max Planck Institute for Multidisciplinary Sciences, Steinegger completed a postdoctoral fellowship at Johns Hopkins University. He is now an associate professor in the Biology Department at Seoul National University in the Republic of Korea, with a joint appointment to the Interdisciplinary Program in Bioinformatics. His group develops user-friendly, open-source methods for protein sequence and structure analysis. In recognition of his scientific contributions, Steinegger received the prestigious 2024 Overton Award from the International Society for Computational Biology.

In this interview with NSR, Steinegger shares his unconventional academic journey, his experiences in developing these transformative tools and his insights into the rapidly evolving field of computational biology.

## Teaser

Martin Steinegger, who is the only non-DeepMind-affiliated author of the AlphaFold2 *Nature* paper, offers unique insights and personal reflections.

## STEINEGGER'S STORY BEFORE AlphaFold

***NSR:*** In the introductory video on your lab's homepage, you mentioned working at a consulting company in 2010 before beginning your undergraduate studies at the Technical University of Munich. This seems to be quite an unconventional path into academia. Could you share the story?

***Steinegger:*** Yes, my path into academia was definitely not traditional. I went to *Hauptschule*, which is a vocational-track school in Germany that doesn't qualify you for university entrance. But my passion for computers pushed me forward, so I continued my education—first going to Austria and later enrolling in a professional school for business informatics. That eventually earned me the qualifications I needed to apply for university.

Before going to university, I started working full-time as an information technology (IT) consultant at Accenture, where I worked in software engineering, performance optimization and security testing. During that time, my former partner encouraged me to consider university, which planted the seed for the idea. But, as I came from a non-academic household, it wasn't an obvious or natural next step—especially for my parents.

In the end, I decided to give it a try and, looking back, it was one of the best decisions of my life.

**Figure 1:**
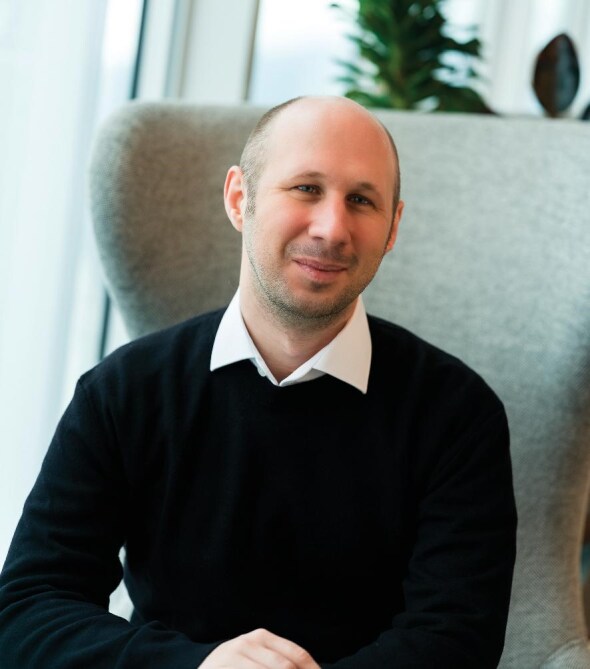
Prof. Martin Steinegger. *(Courtesy of the interviewee)*

***NSR:*** How did you decided to develop MMseqs2, as there already existed widely used sequence search tools such as BLAST?

***Steinegger:*** We originally started with MMseqs(1)—a tool for clustering large protein sequence datasets. We needed this to generate target databases for HHblits—an iterative protein sequence searching tool developed by my PhD group that required multiple sequence alignments for its remote homology searches. At the time, there was no tool fast enough to handle the scale we needed.

[MMseqs2] has evolved into a flexible framework to construct methods that somehow involve searching.

—Martin Steinegger

As we developed MMseqs, we realized that clustering and searching are fundamentally similar tasks—both require comparing many sequences efficiently. However, clustering with MMseqs used global alignments to group similar sequences, whereas search focuses on finding local similarities. By adapting our prefiltering strategy to shifting from global to local alignment, we were able to transform MMseqs into a fast and sensitive sequence search tool—MMseqs2.

Interestingly, even today, many people keep thinking of MMseqs2 as just a clustering tool when, in fact, it is a complete suite for fast protein and profile homology searching.

***NSR:*** Why is MMseqs2 much faster than the old tools?

***Steinegger:*** We applied three main tricks. First, we used an index that maps short words (k-mers) to their positions in sequences, enabling fast lookup. Second, unlike BLAST, which uses short 3-mers by default, we used longer k-mers for better specificity. To avoid losing sensitivity, we allowed mismatches and generated many similar k-mers (neighbors) for each query k-mer. One takeaway was that the gain in specificity outweighs the cost of generating more similar k-mers. Third, we developed an efficient method to detect when two similar k-mers occur on the same diagonal—a strong signal for homology. Combined with a lot of low-level engineering tailored to the hardware available at that time, these ideas resulted in a much faster search.

However, as graphics processing units (GPUs) have become widespread, we need to keep adapting. In our recent preprint on MMseqs2-GPU, we found that MMseqs2’s k-mer approach is not well suited to the computational power of GPUs. Instead, exhaustively computing gapless alignments much better exploits the parallel processing capabilities of GPUs, resulting in improved performance on current hardware.

***NSR:*** Does Foldseek and the other tools that you developed later share some common design philosophies with MMseqs2?

***Steinegger:*** Yes, actually MMseqs2 serves as the foundation for most tools. It has evolved into a flexible framework to construct methods that somehow involve searching. One design decision that really paid off was to split MMseqs2 into modular subcommands—like prefilter, align and cluster. We now have >100 of these modules, some of them very niche and others broadly useful. This has made it quite versatile and easy to develop new tools. For example, Foldseek reuses the prefilter module from MMseqs2 but implements its own structural alignment stage. This modularity saved us a lot of time—we didn't have to reinvent the prefiltering step from scratch for Foldseek and instead just run MMseqs2’s prefilter with 3Di sequences, a 3Di substitution matrix and various parameter tweaks. The later structural Smith–Waterman alignment stage has much larger deviations than MMseqs2’s, so it became its own module.

***NSR:*** Finishing your PhD and postdoc research in the Technical University of Munich, what motivated you to join Seoul National University instead of a European institute? Do you have any comments on the differences in research environments between the East and the West?

***Steinegger:*** This is actually a question I get asked very often, and I understand the curiosity—it's indeed uncommon. Out of 54 faculty members in my department, only two are non-Koreans.

I was very lucky during my PhD with Johannes Söding to have had the freedom to work from wherever I wanted. I initially worked remotely from Barcelona in Prof. Cédric Notredame's lab at the Center for Genomic Regulation (CRG). After a year, I felt like exploring a new place. As I had already moved around quite a bit in the West—Germany, Spain, the USA and Austria—I decided to go East. I was simply curious to learn more about the region and to be out of my comfort zone.

I was fortunate that Prof. Seok Chaok enabled me to stay in Korea. Initially, I planned to stay for just 1 year, but I ended up staying for 3. That experience left a strong impression on me and, when the opportunity at Seoul National University came up, I already knew I liked the environment so I gave it a try!

As for differences in the research environments, one major factor is the fast pace of society and the high value of education. As a consequence, the scale of investment—Korea puts a lot of resources into research and development—is immense and you can really feel the momentum. Also, students here are incredibly bright and motivated, far beyond what I had previously experienced, and I had been already privileged to work with extremely talented people before.

Of course, being a minority in the East comes with its own set of challenges and there are cultural differences to navigate. But, overall, the experience has been highly rewarding, both personally and professionally.

## AlphaFold2 AND ITS IMPACT

***NSR:*** What's your contribution in the development of AlphaFold2? How do you feel about collaborating with DeepMind?

***Steinegger:*** I contributed to the homology retrieval stage, particularly by helping to develop the BFD (Big Fantastic Database), which consists of billions of protein sequences from metagenomes. AlphaFold2 requires a multiple sequence alignment with some diverse sequences to make accurate predictions and BFD played a key role—especially for the hard targets, like viral proteins, in which only very few or no useful hits could be found in public databases at the time. To be honest, when the CASP14 (the challenging 14th Critical Assessment of protein Structure Prediction) results were announced, I couldn't believe what the system had achieved. It was such an inspiring and exciting moment—it felt as though I was witnessing a turning point for the entire field. John [John Jumper, 2024 Nobel Laurent] and the team truly worked machine-learning magic.

***NSR:*** ColabFold is your flagship tool in recent years. It combines the strengths of AlphaFold2 and RoseTTAFold, and provides a convenient entry to these advanced tools. Could you share the story of its development?

***Steinegger:*** ColabFold was actually the result of a few happy coincidences. On the first Sunday after the AlphaFold2 paper (actually the unedited advance access version) was released, I started playing with the source code and ran some predictions for a project. Even though many of the required databases were hosted by us, downloading and setting everything up was a pain—and, like most biology researchers, I didn't have easy access to GPUs.

At around that time, I saw Sergey Ovchinnikov post on Twitter that he was building a Colab notebook that could run AlphaFold2 by using just a single sequence and omitting the multiple sequence alignments (MSAs), directly in the browser via Google Colaboratory. Coincidentally, Milot Mirdita and I had already built and maintained an MMseqs2-based MSA server for Prof. Burkhard Rost's PredictProtein server. So I thought: why not combine Sergey's notebook with our MSA server?

I called Milot and reached out to Sergey; a few hours later—at around 4 a.m. in Korea—we had the first version of ColabFold running. We shared it with the world and, by the next morning, we already had thousands of users and were desperately putting out fires. For instance, most of the system was already running from RAM, but we had forgotten a few very minor file-system accesses to load the protein reference databases, which quickly threatened to overwhelm the storage system.

Since then, we have significantly improved the MSA generation, built many AlphaFold2 hacks into our notebook and set up a dedicated ColabFold server, which has now processed >40 million requests—more than 60 000 per day. This server isn't just the backbone of ColabFold anymore; it now also powers next-generation tools such as Boltz-1, Chai-1, BioEmu and many other models that rely on fast and scalable MSA generation.

···a few hours later—at around 4 a.m. in Korea—we had the first version of ColabFold running.

—Martin Steinegger

***NSR:*** How would you evaluate the revolutionary impact of AlphaFold2 and other AI tools on structural biology research? How could traditional structural biologists face the challenges and opportunities?

***Steinegger:*** AlphaFold2 wasn't just a shock to experimentalists—it was also a shock to developers of protein structure prediction tools. Many thought they had lost their livelihood overnight. It changed the game: suddenly, you could get high-quality structural models in minutes whereas, before, it often took months or even years to solve a crystal structure.

But, at the end of the day, AlphaFold2 is still a hypothesis generator—it needs experimental validation and, for computational method developers, these models allowed an entirely new class of tools to be built, from structural search to protein design.

In practice, most experimental and computational structural biologists whom I have met have fully embraced this paradigm shift. They have found ways to integrate AlphaFold2 into their research and many say that it has made their work easier and more productive.

## INSIGHTS INTO BIOINFORMATICS

***NSR:*** What do you see as the biggest challenges currently facing the field of bioinformatics, especially in protein structure prediction and sequence analysis?

***Steinegger:*** One big challenge is that, while structure prediction has made huge leaps, functional annotation is still lagging behind. In our work on clustering the structural protein universe, we have encountered many protein groups with thousands of members—conserved across all three superkingdoms—yet we have no idea what they do. Not even a clue. This shows how far behind we still are in understanding function.

We also need to go beyond only AlphaFold2 structure predictions, which have impressive accuracy, especially for protein monomers, but it struggles with capturing the effect of small changes—like single-point mutations. Being able to predict the impact of such mutations would open up exciting possibilities in protein optimization, drug design and understanding disease mechanisms.

We need progress both in computational function prediction and in experimental methods for determining function at scale.

***NSR:*** Many of your tools are open-sourced. What is your perspective on the role of open-source tools in scientific research?

***Steinegger:*** Open-source availability is absolutely essential to scientific progress. It allows others to build on your work, validate it and apply it in new ways that you might not have imagined. Many of our tools—MMseqs2, Foldseek, ColabFold—only had the impact they did because they were open and they simply wouldn't exist in their current form without open-source code from others. I really want to thank everyone who open-sources their code—the importance of that can't be overstated.

Open-source availability is also a way to get feedback before peer review. Since ColabFold, we have started the habit of developing all our methods in the open from the beginning. That means that people can benefit from them as early as possible—even before publication—and we receive valuable feedback early on, which often improves the published version.

***NSR:*** How important is international collaboration in your research? Could you share any memorable collaborative experiences?

***Steinegger:*** Most of our major projects are collaborative and I genuinely love to collaborate. It's one of the best ways to learn something new—from people who are experts in areas in which I'm not. Some of the best ideas come out of these exchanges.

One memorable collaboration occurred during the origin of ColabFold, on which I have elaborated already. It started with a tweet from Sergey Ovchinnikov in the USA, my running AlphaFold2 code in Korea and Milot Mirdita jumping in to connect our MSA server from Germany. Within a few hours—across three widely different time zones—we had something working and shared it with the world.

## PERSONAL PERSPECTIVES AND ADVICE

***NSR:*** What were the biggest technical challenges that you faced while developing the tools and how did you overcome them?

***Steinegger:*** A lot of time actually goes into software design—thinking carefully about how to structure tools so that other researchers can use them easily and integrate them into their workflows. Making a tool fast is one thing, but making it usable and reliable at scale is where most of the effort goes.

During my PhD, I spent an unreasonable amount of time thinking about something as simple as how to efficiently find duplicate integers in an unsorted list. It sounds trivial, but optimizing that was a make-or-break issue for MMseqs2. I still find it fascinating how much thought can go into solving such a seemingly small problem—and how those solutions end up enabling large-scale, high-impact tools.

***NSR:*** What are your current research priorities? What exciting research plans do you have for the coming years?

***Steinegger:*** I am especially excited about the intersection of protein structure and metagenomics. In metagenomics, we have long struggled with annotating protein sequences due to their high divergence. But, with the rise of structure prediction tools, we can now find similarities that were previously undetectable at the sequence level. This opens up entirely new ways to explore biology.

We are thinking a lot about how to combine classical bioinformatics algorithms with multimodal language models to improve annotation quality. Our goal is to build tools that can analyse billions of sequences and structures both quickly and accurately—closing the annotation gap and uncovering biology hidden in metagenomic data.

A big part of our mission is also to make these tools widely accessible. That is why we are building user-friendly web servers and interfaces to lower the barrier for researchers around the world to benefit from these advances.

***NSR:*** What advice would you give to young bioinformatics researchers? How should they choose their research directions and tools?

***Steinegger:*** Work on problems that genuinely excite you, even if they seem small (like finding duplicates in an unsorted list). Don't be afraid to take a different path (like starting a group in the Republic of Korea). And share your work early; the feedback will help you to grow faster.
